# *Lavandula stoechas* essential oils protect against Malathion-induces reproductive disruptions in male mice

**DOI:** 10.1186/s12944-018-0891-5

**Published:** 2018-11-08

**Authors:** Slimen Selmi, Kais Rtibi, Dhekra Grami, Hichem Sebai, Lamjed Marzouki

**Affiliations:** Laboratory Functional Physiology and Bio-resources Valorisation, Higher Institute of Biotechnology of Beja, University of Jendouba, Avenue Habib Bourguiba, BP, 382, 9000 Beja, Tunisia

**Keywords:** Malathion, Mice, Steroidogenesis, LSEO, Oxidative stress, StAR gene

## Abstract

**Background:**

The current study was conducted to evaluate the protective effect of *Lavandula stoechas* essential oils (LSEO) against malathion (M) exposure-caused reprotoxicity in male mice as well as the possible mechanisms implicated in such protection.

**Methods:**

Six–eight-week-old male mice weighting 25–30 g were used and divided into four groups: normal-control, LSEO (50 mg/kg, b.w.), malathion (200 mg/kg, b.w.) and malathion + LSEO treated mice. Malathion was emulsioned in corn oil and *per orally* administered for 30 days. LSEO was daily administrated during the same period. LSEO chemical identification was done by Gas chromatography–mass spectrometry (GC-MS). Reproduction-damages and LSEO-benefits were assessed using histopathological, biochemical and steroidogenesis gene expression disruptions and improvements.

**Results:**

The GC-MS analysis, allowed to the identification of 25 bioactive compounds in MCEO. In vivo, we firstly found that malathion exposure induced a clear reprotoxicity as assessed by a significant-decrease (*P* < 0.05) of testis/epididymis relative weights, serum testosterone level and reproductive performance. Malathion also produced lipoperoxidation, thiol (-SH) groups decrease as well as a significant-depletion (*P* < 0.05) of antioxidant enzyme activities such as catalase (CAT) and glutathione peroxidase (GPx), total superoxide dismutase (SOD), Cu/Zn-SOD and Mn-SOD in testis and epididymis. The histopathological examination showed marked change in both studied tissues. All these biochemical and structural changes were significantly (*P* < 0.05) corrected by LSEO co-administration. More importantly, malathion exposure remarkably (*P* < 0.05) down-regulated the expression of StAR gene as well as, the mRNA levels of P450scc, 3ßHSD and 17ß-HSD, while LSEO-administration strangely protected against steroidogenesis disruption.

**Conclusions:**

The potential protective effects of LSEO against malathion-induced reprotoxicity and oxidative stress might be partially to its antioxidant properties as well as its opposite effect against some gene expression involved in the steroidogenesis.

## Background

Some organophosphorus (OPs) compounds are known by their effects on impair fertility, suppressif libido, causif testicular degeneration, and deterioration in semen quality [1]. The malathion is an organophosphorus pesticide extensively used as a systemic insecticide and acaricide, which was proved to be neurotoxic and to disturb StAR gene expression [1, 2]. In fact, in our earlier study, we have shown that continual exposure to malathion decreases testicular weight, serum testosterone level, sperm motility, and increases the percentage of dead and abnormal sperm in male mice [3, 4]. As spermatogenesis and steroidogenesis are dependent upon the maintenance of adequate levels of testosterone and cholesterol, the ability of malathion to reduce their levels may contribute to the reduction of male mice reproductive capability [1]. In fact, several authors have revealed that diazinon inhibit steroidogenesis in the testis [5, 6]. However, various naturally occurring compounds are largely used to protect against reproductive damage and many pathways complications in both the experimental and medical situations. For this reason, the World Health Organization (WHO) has established a program for the use of traditional herbal medicines in the treatment of diverse diseases difficulties and complications [7].

*Lavandula stoechas* is a well identified medicinal plant species in the word, including Tunisia. However, due to its high content in therapeutically active compounds, this plant presents many beneficial health properties in part due to its antioxidant and anti-inflammatory actions [8]. Recently, it has been demontrated in our laboratory that *Lavandula stoechas* essential oils (LSEO) have the capacity to reduce blood glycaemic level and protect against oxidative stress induced by alloxan in rats [9]. Additionally, it has been reported that the LSEO supplementation especially protects against the perturbation of lipid metabolic parameters induced by this insecticide intoxication in rats [10].

In our knowledge, the toxicity of malathion on testicular Leydig cell function is still unknowned. Therefore, in the current study we intended to investigate the action of a sub-chronic exposure of male mice to malathion on Leydig cell steroidogenesis, free radicals production as well as StAR, P450 scc, 3β-HSD and 17β-HSD genes expression. In addition, this study aims also to investigate the protective effect of *Lavandula stoechas* essential oils (LSEO) against all damages induced by this insecticide as well as the mechanisms involved in this protection.

## Methods

### Chemicals

Malathion (Fyfanon EC 500) was obtained from INNOVA Society size in Tunis, Tunisia at The purity of 96%. 2-Thio-barbituric acid (TBA), bovine catalase, Epinephrine, and butylated hydroxytoluene (BHT) were from Sigma aldrich chemicals Co (Germany). All other chemicals used were of analytical grade.

### Plant collection

The aerial parts of *Lavandula stoechas* were collected from the area of Ain-Draham (North-West of Tunisia) and identified in laboratory of taxonomy in the Faculty of Sciences of Tunis (FST)-Tunisia. The Voucher specimens (No. L101) have been deposited with the herbarium of the Higher Institute of Biotechnology of Beja, Tunisia.

### Plant extracts preparation

Plant extracts preparation was performed according as describe by Selmi et al. [10].

### Gas chromatography-mass spectrometry (GC-MS)

The essential oils of *L. stoechas* were subjected to GC-MS analysis using Trace GC ULTRA /Polaris Q (GC-MS, Thermo Electron) as described by Sebai et al. with a slight modifications [9].

### Animals treatments

Adult male mice (25–30 g) and were cared for in compliance with the code of practice for the Care and Use of Animals for Scientific Purposes. Approval for these experiments was obtained from the Medical Ethical Committee for the Care and Use of Laboratory Animals of Pasteur Institute of Tunis (approval number: LNFP/Pro 152,012). The experimental protocols were approved by the Faculty Ethics Committee (Faculty of Sciences, Tunis, Tunisia). The animals were housed in standard cages (40 × 28 × 16 cm) under controlled conditions: 12:12-h light:dark, 20–22 °C, food and water are ad libitum. Mice were after divided into 4 groups of 12 animals in each:

Group I (CTR): animals served as control and received equivalent volume of corn oil (1 ml), group II (M): animals were treated with malathion at 200 mg/kg body weight/day dissolved in 1 ml of corn oil, group III (LSEO): animals received *Lavandula stoechas* essential oils (50 mg/kg b.w.) and group IV (M + LSEO): animals received both malathion and LSEO under the same conditions.

### Body and reproductive organs weights

Initial (weight at the starting point) and final (weight at the end point) body weights were recorded. Testis and epididymis were stripped from fatty tissues and blood vessels, blotted, and their absolute weights were determined. Clinical signs of body and reproductive organs were evaluated for toxicological criteria. To normalize the data for statistical analysis and to obtain relative weight, data were expressed per 100 g body weight.

### Testosterone determination

Serum testosterone level was quantified by enzyme-linked immunosorbent assay (ELISA) using specific commercial kits (DEMEDITEC Diagnostics GmbH, Germany). Data were determined according to the manufacturer’s protocols as described by Selmi et al. [3].

### Evaluation of sperm characteristics

#### Sperm collection

Sperm collection was performed as described by Selmi et al. [3]. The sperm count was assessed from right cauda epididymis, whereas sperm motility and morphology were analyzed from the left one.

#### Sperm count, motility, viability and morphology

The cauda epididymal sperm count, motility, viability and morphology was performed as described by Selmi et al. [3] according successively to the methods of Vega et al. [11], Kvist and Bjorndahl [12], Tardif et al. [13], WHO [14], Seed et al. [15] and Filler [16].

### Steroidogenesis gene expression analysis

RNA extraction and RT-PCR Total RNA from rat testis was isolated with RNeasy Mini Kit (ambion by Life Technology) according to the manufacturer’s protocol as described by Selmi et al. [3]. The relative mRNA abundance was calculated by the ratio of sample-to-control.

### Oxidative stress biomarkers

#### Lipid peroxidation and H2O2 generation

Lipid peroxidation was detected by the determination of MDA content as described by the method of Buege and Aust [17] with a slight modification. MDA levels were determined by using an extinction coefficient for MDA-TBA complex of 1.56 10^5^ M^− 1^ cm^− 1^ and expressed as nmol/mg protein. Hydrogen peroxide content in both reproductive-organs was performed according to Dingeon et al. [18] and the results were expressed as μmol of H_2_O_2_ per mg of protein.

#### Thiol groups measurement

Total Thiol groups concentration (-SH) was performed according to Ellman method with slight modifications [19]. Results were expressed as nmol of thiol groups per mg of protein.

#### Antioxidant activities assays

Determination of SOD activity was performed according of Misra and Fridovich with slight modifications [20]. Characterization of SOD isoforms was performed using KCN (2 mM), which inhibits Cu/Zn-SOD or H_2_O_2_ (5 mM), affecting both Cu/Zn-SOD and Fe-SOD whereas Mn-SOD was insensitive to both inhibitors [21].

CAT activity was assayed according to Aebi with slight modifications [22]. CAT activity calculated using the extinction coefficient of 40 mM^− 1^ cm^− 1^ for H_2_O_2_.

GPx activity was determined according to Flohé and Günzler [23]. GPx activity was expressed as nmol of GSH consumed/min/mg protein.

### Protein determination

Protein content in the testis and epididymis was determined by the method of Bradford using bovine serum albumin (BSA) as standard [24].

### Histopathological examination

Histopathological examination in testis and epididymis tissues was performed by the method described by Selmi et al. [3].

### Statistical analysis

The data were analyzed by unpaired *t-*student test and were expressed as means ± standard error of the mean (S.E.M.). The data are representative of 12 independent experiments. All statistical tests were two-tailed, and a *p* value of 0.05 or less was considered significant.

## Results

### Chemical composition of the lavender essential oils

*L. stoechas* essential-oil bioactive-compounds were identified by the GC–MS technique and presented in Table 1. In this respect, thirty-two compounds have been identified. The main components are: D-Fenchone (29.28%), α-pinene (23.18%) and camphor (15.97%). These cyclic compounds belong essentially to the family of oxygenated monoterpenes known for their antioxidant and free radical scavengers.

**Table 1:** *L. stoechas* essential-oil phytochemical composition identified using a GC–MS analyze

No	Components	IR	Compositions (%)
1	Tricyclene	6.137	0.51
2	α-pinene	6.720	23.18
3	Camphene	7.310	7.83
4	β-Phellandrene	8.500	0.10
5	β -Pinene	8.626	0.12
6	Delta 3-Carene	10.554	0.11
7	Cymene	11.504	0.72
8	Limonene	11.807	2.71
9	Eucapur	11.893	3.29
10	D-Fenchone	15.835	29.28
11	Linalool	16.785	2.01
12	Camphor	19.526	15.97
13	Myrtenol	23.228	0.26
14	Endobornyl Acetate	29.122	1.03
15	Aromad Endrene	33.991	0.28
16	α -Copaene	34.809	0.28
17	Caryophyllene	37.436	0.26
18	β -Selinene	41.533	0.26
19	Delta-Cadinene	43.953	0.67
20	α -Elemene	44.434	0.12
21	Selina-3,7(11)-diene	44.817	0.85
22	Delta-gurjunene	47.730	0.20

*IR* Retention Index

### Testis and epididymis relative weights

Data from Table 2, showed that sub-chronic exposure of male mice to malathion-induced a significant decrease (*P* < 0.05) of testis/epididymis relative-weights. Indeed, these last were reduced from 0.52 ± 0.01 to 0.63 ± 0.06 and from 0.09 ± 0.01 to 0.12 ± 0.02 in malathion-treated-group respectively. However, LSEO pre-treatment significantly (*P* < 0.05) protected against malathion-caused reproductive organs weight loss.

**Table 2:** Effect of sub-chronic treatment (30 days) malathion (200 mg / kg BW, po) and / or essential oils of lavender (50 mg / kg, PC, OP) on relative weight of testis and epididymis in male mice

Parameters	CTR	M	LSEO	M + LSEO
Testis relative weight (g/100 g b.w)	0.52 ± 0.01	0.63 ± 0.06*	0.54 ± 0.04^*#*^	0.49 ± 0.02^*#*^
Epididymis relative weight (g/100 g b.w)	0.09 ± 0.01	0.12 ± 0.02*	0.09 ± 0.02^*#*^	0.1 ± 0.02^*#*^

The results represent the mean ± SEM (*n* = 12). (*: *P* < 0.05 vs. control, and #: *P* < 0.05 vs malathion-group by the *student-test*)

### Testosterone determination

**Fig. 1:**
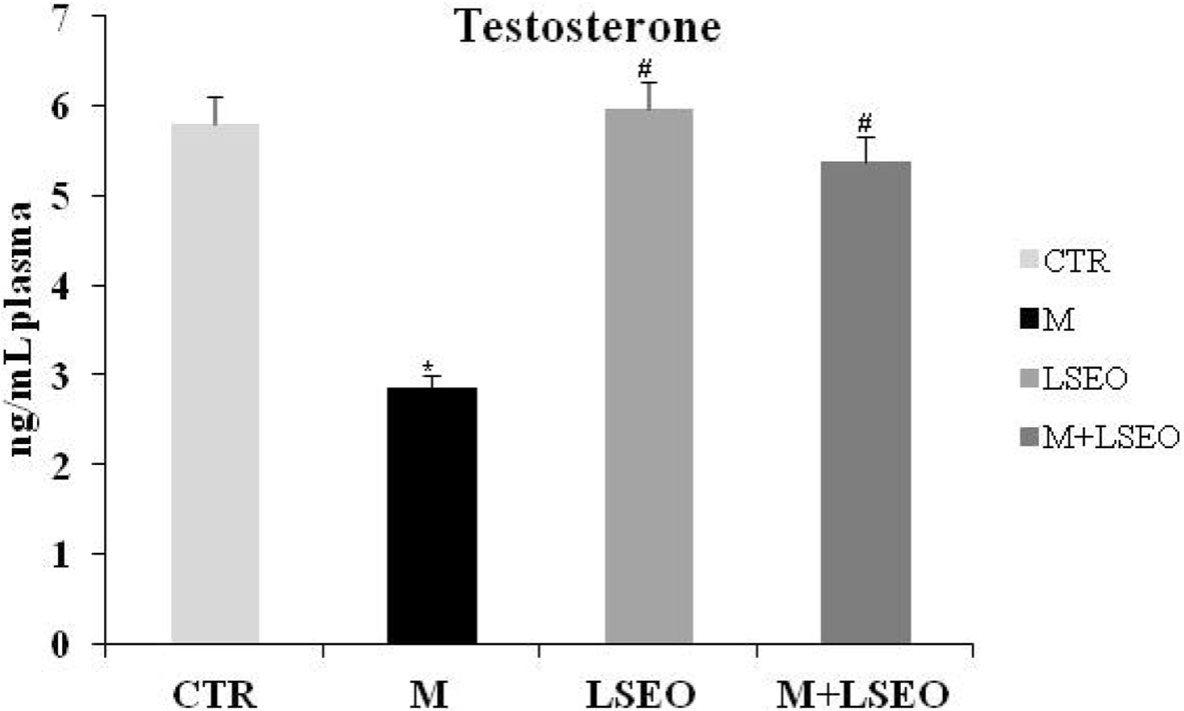
Subchronic effect (30 days) of malathion (200 mg / kg BW, po) and / or lavender essential oils (LSEO) (50 mg / kg, PC, OP) on testosterone levels in male mice. Values are expressed as mean ± SEM(*n* = 12). * Comparison of CTR and other groups (*p* 0.05). ^*#*^ Comparison of **M** with **LSEO** group (*p* 0.05)

Data from Fig. 1**,** showed that sub-chronic exposure of male mice to malathion-induced a significant diminution (*P* < 0.05) of serum testosterone level in all treated-groups. In contrast, the Co-administration of malathion and LSEO had no effect on the testosterone level.

### Evaluation of reproductive performance quality

**Fig. 2:**
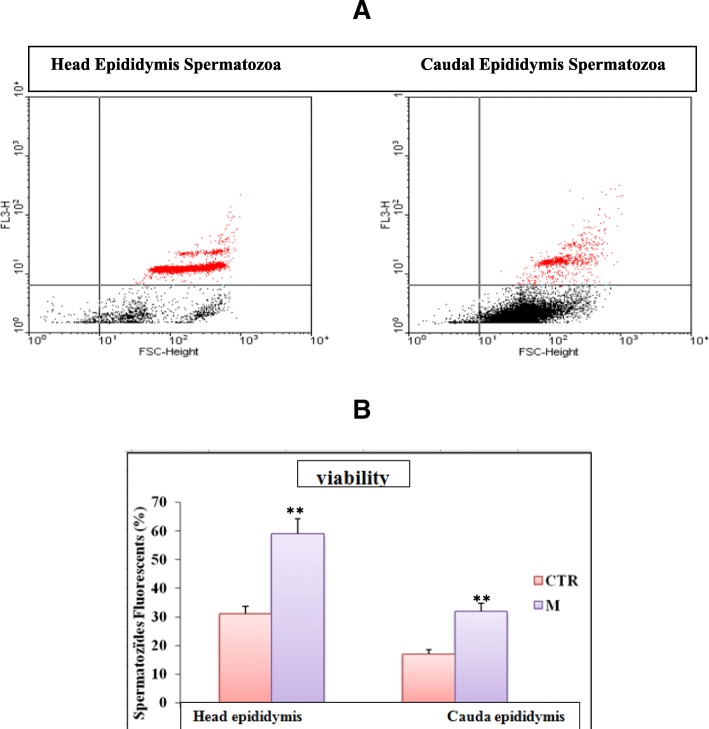
Determination of sperm viability by flow cytometry using propidium iodide (PI) as a probe. **a** Chart type obtained with the cytometer shows the dispersion of the cells according to their fluorescence (FL3) and size (FSC). The fluorescence threshold is indicated, the red dots placed above this threshold represent the fluorescent cells. **b** The percentage of fluorescent sperm is then determined by the software and averages are calculated is presented as a histogram (*n* = 6). ** *P* < 0.01 Control vs Malathion

**Fig. 3:**
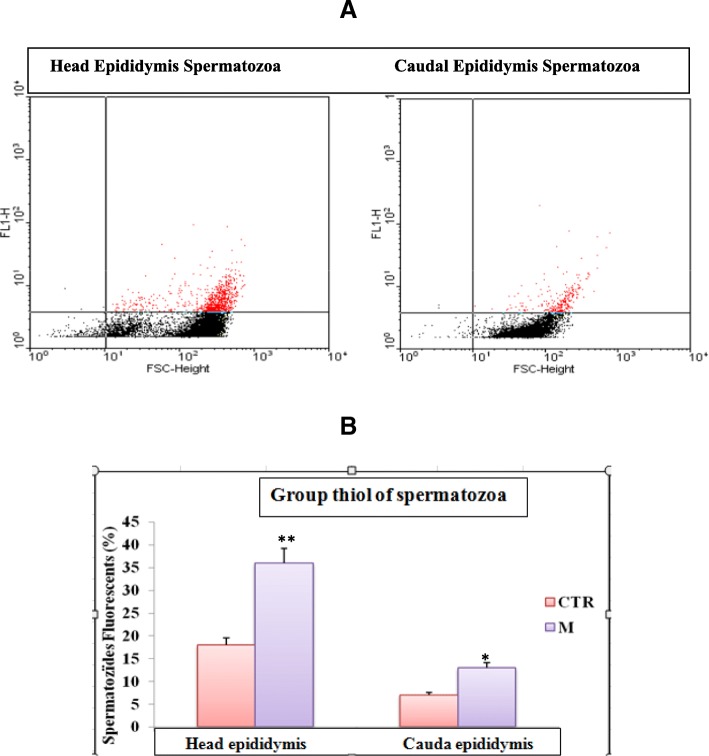
Determination of spermatic thiols groups by flow cytometry using monobromobimane (MB) as a probe. **a** Chart type obtained with the cytometer shows the dispersion of the cells according to their fluorescence (FL3) and size (FSC). The fluorescence threshold is indicated, the red dots placed above this threshold represent the fluorescent cells. **b** The percentage of fluorescent sperm is then determined by the software and averages are calculated is presented as a histogram (*n* = 6). ** *P* < 0.01 Control vs Malathion

**Fig. 4:**
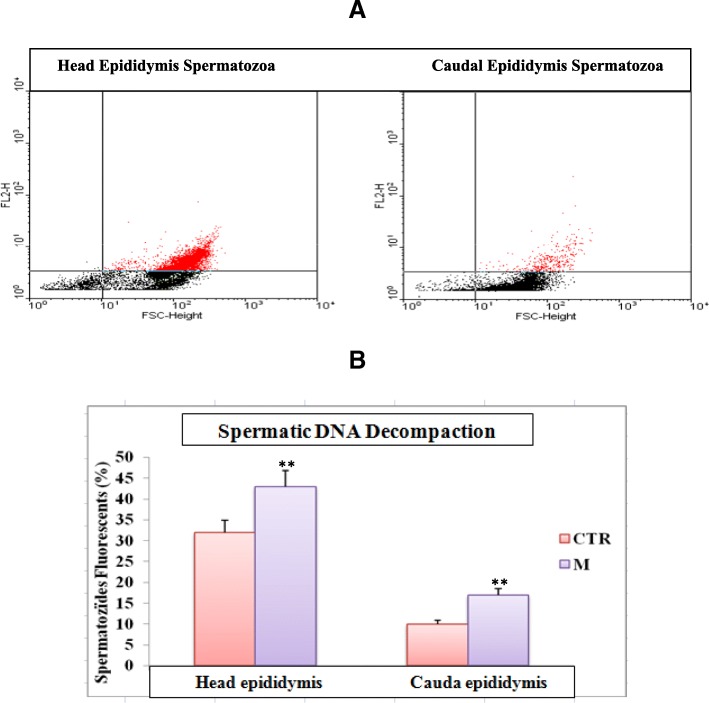
Determination of spermatic DNA decompaction by flow cytometry using chromomycine A3 (CMA3) as a probe. **a** Chart type obtained with the cytometer shows the dispersion of the cells according to their fluorescence (FL3) and size (FSC). The fluorescence threshold is indicated, the red dots placed above this threshold represent the fluorescent cells. **b** The percentage of fluorescent sperm is then determined by the software and averages are calculated is presented as a histogram (*n* = 6). ** *P* < 0.01 Control vs Malathion

Data from Table 3, showed that 30 days exposure to malathion-provoked a significant depletion (*P* < 0.05) of different sperm parameters such as epididymal sperm concentration (7.36 ± 2.00 to 2.76 ± 1.00 10^6^/mL), motility (69.00 ± 7.00% to 40.00 ± 5.00%), viability (79.00 ± 4.00% to 22 .00 ± 6.00%), sperm count (132.00 ± 16.40 to 96.00 ± 1.98 10^6^/g epididymis) and testicular spermatids count (200.00 ± 9.30 to 118.00 ± 5.89), while, the number of dead sperm increased remarkably (*P* < 0.05) (Table 3). Added to that, malathion-produced a significant decrease of viability, thiols-groups and DNA-decompaction as assessed by flow cytometry using monobromobimane (MB), propidium iodide (PI), chromomycine A3 (CMA3) as a probe (Figs. 2, 3 and 4).

**Table 3:** Effect of sub-chronic treatment (30 days) malathion (200 mg / kg BW, po) and / or essential oils of lavender (50 mg / kg, PC, OP) on variation in the sperm parameters in male-mice

Parameters	CTR	M	LSEO	M + LSEO
Sperm concentration (10^6^/mL)	7.36 ± 2.00	2.76 ± 1.00*	7.89 ± 1.00^*#*^	7.21 ± 2.00^*#*^
Motility (%)	69.00 ± 7.00	40.00 ± 5.00*	68.00 ± 4.00^*#*^	67.00 ± 5.00^*#*^
Viability (%)	79.00 ± 4.00	22 .00 ± 6.00*	81.00 ± 4.00^*#*^	72.00 ± 5.00^*#*^
Morphology (normal form) (%)	86.10 ± 4.52	46.00 ± 3.00*	89.00 ± 4.56^*#*^	79.00 ± 5.38^*#*^
Head Abnormal sperm (%)	5.80 ± 0.96	14.00 ± 2.10*	4.62 ± 1.12^*#*^	6.49 ± 1.71^*#*^
Caudal Abnormal sperm (%)	8.10 ± 1.41	39.00 ± 4.20*	11.60 ± 2.30^*#*^	15.80 ± 1.90^*#*^
Sperm count 10^6^/g epididymis	132.00 ± 16.40	96.00 ± 1.98*	134.00 ± 1.96^*#*^	123.00 ± 3.49^*#*^
Spermatid count 10^6^/g of testis	200.00 ± 9.30	118.00 ± 5.89*	189.00 ± 7.21^*#*^	183.00 ± 8.78^*#*^

The results represent the mean ± SEM (*n* = 12). (*: *P* < 0.05 vs. control, and #: *P* < 0.05 vs Malathion by the student t-test)

Morphological abnormalities of spermatozoa were evaluated based on head or tail forms. LSEO co-administration (50 mg/kg) to male mice intoxicated with malathion at a dose of 200 mg/kg leads to a restoration of all male-fertility-parameters and reduce abnormal spermatozoa forms and the results were comparable to those experimental in the normal-animals.

### Steroidogenesis gene expression analysis

**Fig. 5:**
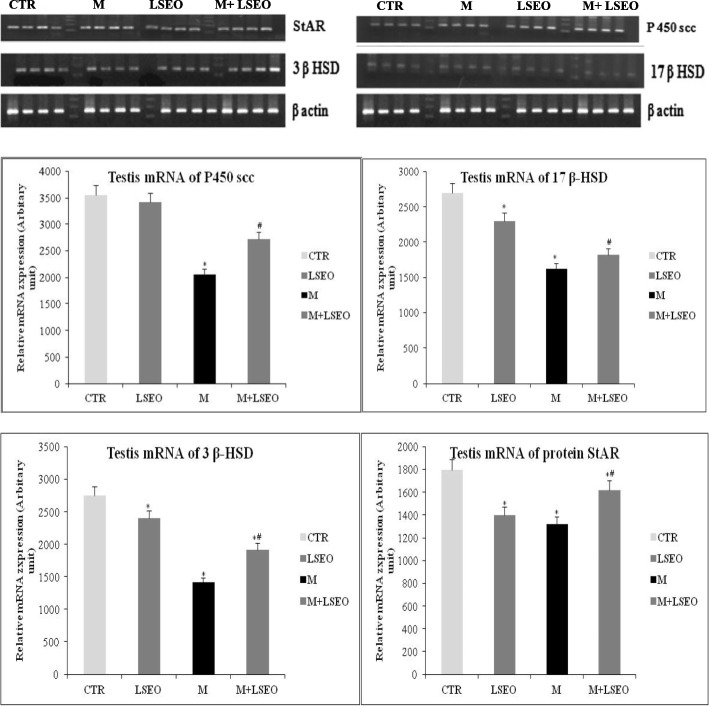
Evaluation of mRNA expressions of enzymes and proteins involved in steroidogenesis.Values are expressed as mean ± SEM(*n* = 12). * Comparison of CTR and other groups (p 0.05). ^*#*^ Comparison of **M** with **LSEO** group (p 0.05)

We further looked at the effect of malathion (200 mg kg, b.w.) exposure during 30 days on StAR protein gene-expression responsible for cholesterol transport as well as steroidogenic enzymes P450scc, 3ßHSD, and 17ß-HSD. The expression levels were normalized by β-actin expression detected in the same PCR reaction, and these values were compared with those of the control group. As expected, there was no change in the expression of β-actin in control and treated groups. However, malathion exposure caused a singnificant-decrease (*P* < 0.05) in both StAR, P450scc, 3ßHSD, and 17ß-HSD when compared to control group. In opposite, LSEO co-administration of had no effect on steroidogenesis gene expression (Table 4 and Fig. 5).

**Table 4:** Primers used for RT-PCR

Primer	Primer Sequence (5′-3′)	Tm (°c)
StAR	Forward: GCCCCGAGACTTCGTAAG Reverse: CAGGTGGGACCGTGTTCA	**59**
P450scc	Forward: GCCCCGAGACTTCGTAAG Reverse: CAGGTGGGACCGTGTTCA	**60**
3β-HSD	Forward: CCTGCTGCGTCCATTTTA Reverse: TCTGCTTGGCTTCCTCCC	**60**
17β-HSD	Forward: ACCGCCGATGAGTTTGTT Reverse: GGGTGGTGCTGCTGTAGA	**60**
β-Actin	Forward: GAGATTACTGCCCTGGCTCCTA Reverse: ACTCATCGTACTCCTGCTTGCTG	**60**

### Oxidative stress biomarkers and antioxidants enzymes activities

To assess the effect of the organophosphorus compound and essential-oils on oxidative stress situation, the some antioxidant biomarkers were evaluated in the different groups. Results showed that oxidative-stress induced by malathion-treatment is explained by a significant increase (*P* < 0.05) of lipoperoxydation in testis and epididymis, hydrogen peroxide production (*P* < 0.05) as well as a decrease (*P* < 0.05) of thiol groups content (Tables 5, 6 and 7). However, the treatment of animals with LSEO protects against all these alterations caused by malathion-intoxication. On another hand, we examined the antioxidant-enzyme-activities in testis and epididymis (Tables 8, 9, 10 and 11). As expected, malathion-intoxication induced a significantly (*P* < 0.05) reduce of CAT, GPx, total SOD and SOD isoforms such as Cu/Zn-SOD and Mn-SOD activities. The sub-chronic co-administration of LSEO markedly (P < 0.05) protected against the depletion of testis/epididymis antioxidant enzyme activities induced by malathion exposure during 30 days.

**Table 5:** Effect of sub-chronic treatment (30 days) malathion (200 mg / kg BW, po) and / or essential oils of lavender (50 mg / kg, PC, OP) on variation of MDA levels on testis and epididymis of male mice

MDA (nmol/mg protein)				
	CTR	M	LSEO	M + LSEO
Testis	3.80 ± 0.37	6.20 ± 0.35*	3.60 ± 0.34	4.10 ± 0.22^*#*^
Epididymis	3.20 ± 0.28	5.30 ± 0.46*	3.20 ± 0.14^*#*^	3.50 ± 0.18^*#*^

The results represent the mean ± SEM (*n* = 12). (*: *P* < 0.05 vs. control, and #: *p* < 0.05 vs Malathion by the student t-test)

**Table 6:** Effect of a sub-chronic treatment (30 days) by malathion (200 mg / kg BW, po) and / or essential oils of lavender (50 mg / kg, PC, OP) on thiols groups level of the testis and epididymis in male mice

-SH (nmol/mg of protein)				
	CTR	M	LSEO	M + LSEO
Testis	0.89 ± 0.04	0.58 ± 0.03*	0.87 ± 0.04^*#*^	0.76 ± 0.03^*#*^
Epididymis	0.36 ± 0.02	0.26 ± 0.02*	0.37 ± 0.02^*#*^	0.37 ± 0.02^*#*^

The results represent the mean ± SEM (*n* = 12). (*: *P* < 0.05 vs. control, and #: *p* < 0.05 vs Malathion by the student t-test)

**Table 7:** Effect of sub-chronic treatment (30 days) malathion (200 mg / kg BW, po) and / or essential oils of lavender (50 mg / kg, PC, OP) on variation of the rate of hydrogen peroxide (H_2_O_2_) in testis and epididymis in male mice

H_2_O_2_ (μmol/mg proteins)				
	CTR	M	LSEO	M + LSEO
Testis	0.84 ± 0.07	1.78 ± 0.06*	0.88 ± 0.08^*#*^	0.96 ± 0.06^*#*^
Epididymis	0.69 ± 0.06	1.23 ± 0.05*	0.63 ± 0.06^*#*^	0.89 ± 0.02*^#^

The results represent the mean ± SEM (*n* = 12). (*: *P* < 0.05 vs. control, and #: *p* < 0.05 vs Malathion by the student t-test)

**Table 8:** Effect of sub-chronic treatment (30 days) malathion (200 mg / kg BW, po) and / or essential oils of lavender (50 mg / kg, PC, OP) on catalase (CAT) activity of levels in testis and epididymis in mice

Catalase (nmol min^− 1^ mg^− 1^ proteins)				
	CTR	M	LSEO	M + LSEO
Testis	563.00 ± 37.40	246.00 ± 23.90*	578.00 ± 28.50*^#^	594.00 ± 36.20^*#*^
Epididymis	193.00 ± 19.80	98.00 ± 17.60*	196.00 ± 25.40^*#*^	162.00 ± 22.70*^#^

The results represent the mean ± SEM (*n* = 12). (*: *P* < 0.05 vs. control, and #: *p* < 0.05 vs Malathion by the student t-test)

**Table 9:** Effect of sub-chronic treatment (30 days) malathion (200 mg / kg BW, po) and / or essential oils of lavender (50 mg / kg, PC, OP) on glutathione peroxidase (GPx) activity of testis and epididymis in mice

GPx (GSH consumed/min/mg protein)				
	CTR	M	LSEO	M + LSEO
Testis	9.20 ± 0.90	4.70 ± 0.5°*	9.60 ± 0.45^*#*^	8.20 ± 0.47^*#*^
Epididymis	7.50 ± 0.50	3.30 ± 0.60*	7.70 ± 0.20^*#*^	6.70 ± 0.42^*#*^

The results represent the mean ± SEM (n = 12). (*: *P* < 0.05 vs. control, and #: *p* < 0.05 vs Malathion by the student t-test)

**Table 10:** Effect of sub-chronic treatment (30 days) malathion (200 mg / kg BW, po) and / or essential oils of lavender (50 mg / kg, PC, OP) on variation in the activity of superoxide dismutase (SOD) total (U of SOD activity per mg proteins), Cu / Zn SOD, Mn-SOD and Fe-SOD in the testes in mice

Testis SOD	CTR	M	LSEO	M + LSEO
SOD Total	8.34 ± 0.67	4.48 ± 0.42*	8.43 ± 0.38^*#*^	6.89 ± 0.51*^#^
cu/zn-SOD	4.36 ± 0.45	2.23 ± 0.24*	4.97 ± 0.41^*#*^	4.09 ± 0.3^*#*^
Mn-SOD	2.49 ± 0.36	1.47 ± 0.15*	2.56 ± 0.13^*#*^	1.63 ± 0.27*
Fe-SOD	1.14 ± 0.13	1.09 ± 0.18	1.16 ± 0.22	1.18 ± 0.12

The results represent the mean ± SEM (n = 12). (*: *P* < 0.05 vs. control, and #: *p* < 0.05 vs Malathion by the student t-test)

**Table 11:** Effect of sub-chronic treatment (30 days) malathion (200 mg / kg BW, po) and / or essential oils of lavender (50 mg / kg, PC, OP) on variation in the activity of superoxide dismutase (SOD) total (U of SOD activity per mg proteins), Cu / Zn SOD, Mn-SOD and Fe-SOD in the epididymis in mice

Epididymis SOD	CTR	M	LSEO	M + LSEO
SOD Total	6.67 ± 0.47	3.56 ± 0.28*	6.63 ± 0.38^*#*^	6.29 ± 0.51^*#*^
cu/zn-SOD	3.91 ± 0.22	1.21 ± 0.22*	3.64 ± 0.23^*#*^	3.82 ± 0.12^*#*^
Mn-SOD	1.62 ± 0.16	1.42 ± 0.21*	1.66 ± 0.11^*#*^	1.69 ± 0.09^*#*^
Fe-SOD	0.78 ± 0.07	0.71 ± 0.05	0.74 ± 0.06	0.76 ± 0.04

The results represent the mean ± SEM (*n* = 12). (*: *P* < 0.05 vs. control, and #: *p* < 0.05 vs Malathion by the student t-test)

### Histopathological examination

**Fig. 6:**
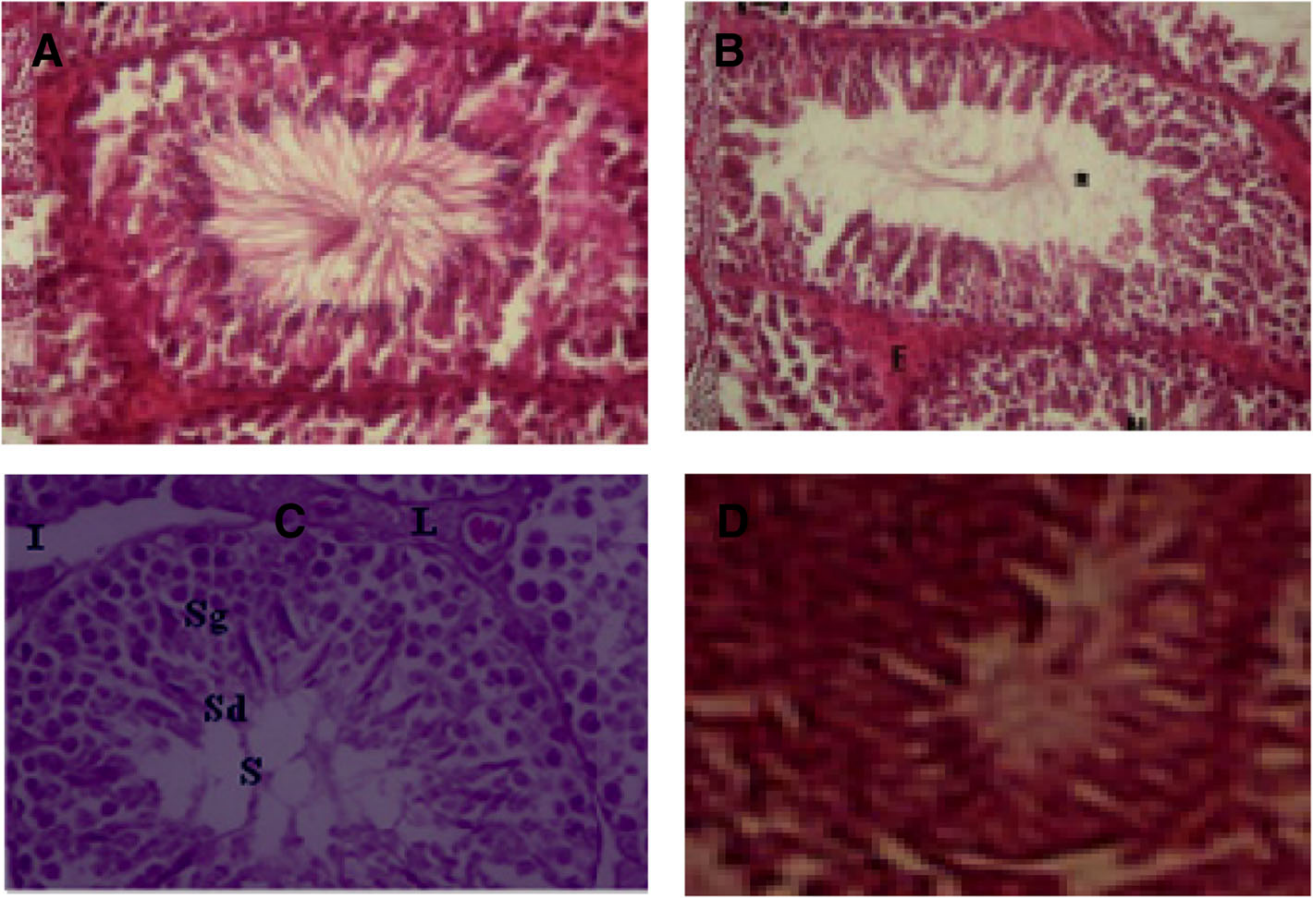
Histological changes induced by subchronic malathion exposure (200 mg/kg/b.w., p.o.) during 30 days (**b**), Normal architecture in control group (**a**), LSEO group (**c**) and coadministration of M+ LSEO (**d**); (X400). L: leidigs cells; I: Interstitial space; Sg: Spermatogonia; Sd: Spermatid; S: Spermatozoa; *: decrease of spermatozoa density

**Fig. 7:**
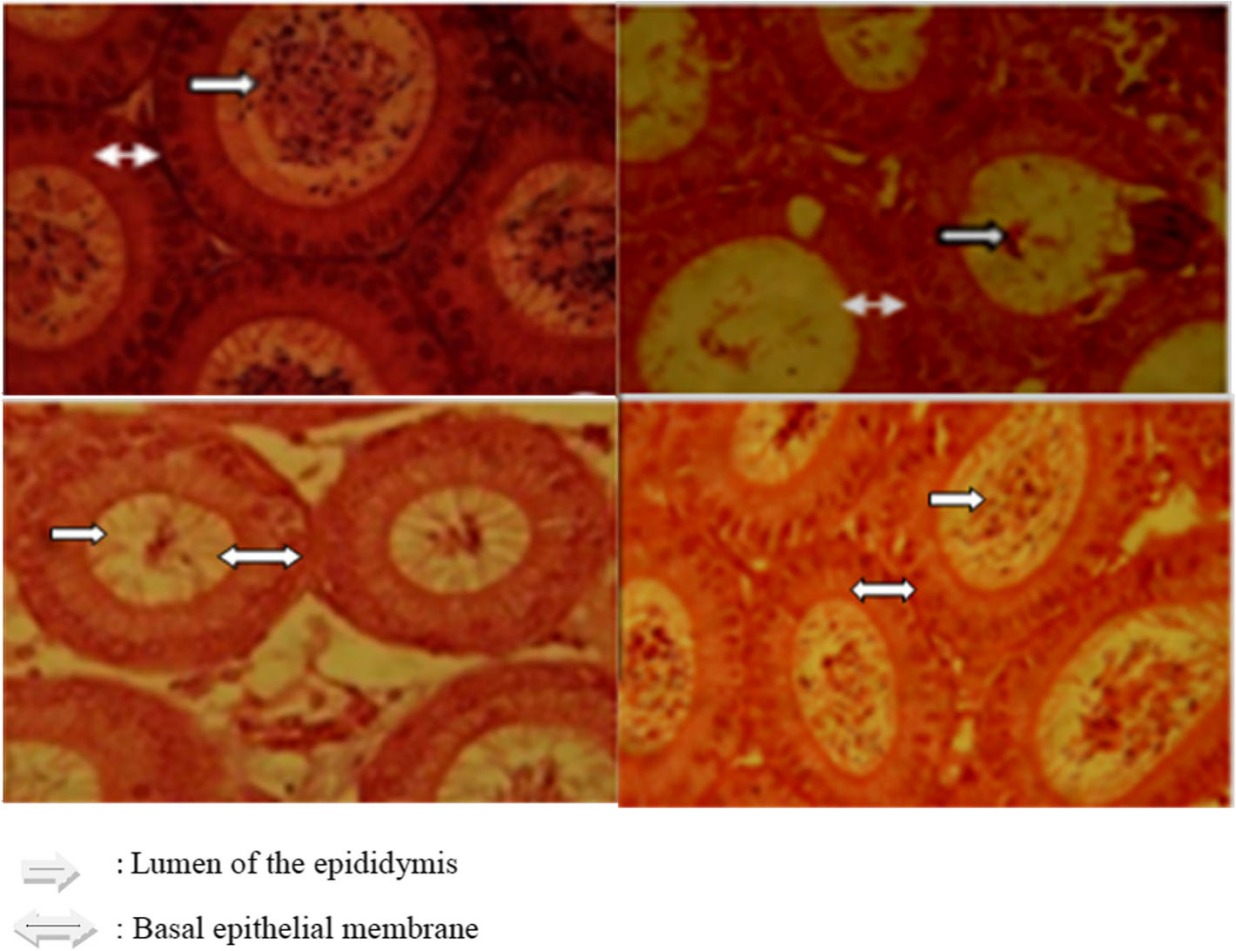
Histomorphology of epididymis observed under light microscope (400 magnification). (**a**) Control, (**b**) Malathion treated, (**c**) LSEO, (**d**) M + LSEO. : Lumen of the epididymis.: Basal epithelial membrane

Histological organization of the testis is illustrated in Fig. 6**.** Testis of undamaged control (Fig. 6a) showed regular seminiferous tubules lined with germinal epithelial layer. Different types of spermatogenic, cells appeared in their ordinary form with: spermatogonia, primary and secondary spermatocytes, spermatids and mature spermatozoa. Sertoli and Leydig cells with standard form were observed. Leydig cells and blood vessels were found in the interstitial connective tissues between the seminiferous tubules, and the tubules which appeared with regular size and shape. Malathion exposure (Fig. 6b) caused necrosis, deterioration, diminishing the number of spermatogenic cells in seminiferous tubules, unscrambling of cells from basal region of seminiferous tubules and loss the Leydig cells in interstitial tissue. LSEO Co-administrated to malathion protected and recovered the damages of testicular tissues, induced by the malathion intoxication. On the other hand, concerning the histomorphology of epididymis, we showed that malathion exposure caused a loss of sperm from the lumen of epididymis as well as the disintegration of basal membrane (Fig. 7b), whereas the co-treatment with LSEO restored the architecture of epididymis as evidenced by the accumulation of sperm in the centre of lumen and the regeneration of lining cells (Fig. 7c and d).

## Discussion

Firstly, the chemical analysis using GC-MS technique allowed to the identification of 22 compounds. In vivo part study, we showed that the subchronic-exposure to malathion leads to a significant decline of testis/epididymis relative weights indicating a testicular deterioration. Added to that, our results showed a marked decrease in serum testosterone levels in male mice after chemical intoxication. These alterations were accompanied with a concomitant decrease in the mRNA level of the genes involved in the transport and transfer of cholesterol and steroidogenesis, including that of the StAR protein, P450scc, 3β-HSD and 17β-HSD, was observed in contrast to an increase in the total cholesterol level of the same group. More importantly, we showed in this study, a significative-diminution in mRNA expression of StAR, P450scc, 3ßHSD, and 17ß-HSD was detected after malathion-exposure, while, LSEO co-administration extensively ameliorated the mRNA expression of studied genes. We also studied the effect of coadministration of malathion and LSEO on oxidative stress parameters. In this context, we showed that malathion-administration induced oxidative stress as assessed by an increase of lipid peroxidation and hydrogen peroxide level, a decrease of sulfhydrils content, as well as a depletion of antioxidant enzyme activities such as CAT, GPx, total SOD, Cu/Zn-SOD, Mn-SOD, and Fe-SOD in testis and epididymis. All these alterations were associated with histopathological changes in both reproductive-organs.

Testis weight is principally dependent on the mass of the differentiated spermatogenic cells; the lessening in its weight may be due to decreased number of germ cells, inhibition of spermatogenesis, and steroidogenesis [25, 26]. This loss of weight may also be due to a reduction in the bioavailability of testosterone, LH and FSH, whose levels in the bloodstream indicate the reproductive endocrine status of the male [27]. We also found that LSEO treatment significantly attenuated the effect of malathion on testicular weight. However, LSEO is known for its potential for tissue chelation and supplementation during malathion poisoning, and reduces the level of pesticide and its active metabolites in testicular tissue.

Since testicular Leydig cells are the site of synthesis of male steroid hormone. This hormone also plays a key role in the conservation of male sexual characteristics, spermatogenesis and fertility [28]. Our results are in agreement with our previous work, which found that a low serum testosterone concentration in rats may be due to the inhibition of the activities of steroidogenic enzymes [3], which are responsible for regulating the biosynthesis of testosterone, or because of the deleterious effects caused by malathion on Leydig cells through the generation of ROS [29, 30]. Importantly, we showed in this study that LSEO co-administration stimulated the testosterone biosynthesis. This beneficial effect on testosterone defence might be in part due to the protection of Leydig cells beside malathion intoxication by LSEO [31].

However, the conversion of cholesterol to testosterone in Leydig cells, the cholesterol is done in several stages and is under the action of different enzymes for each [32]. The free cholesterol tranfer of the extacellular and intracellular medium to the internal mitochondrial membrane is Limiting stage of steroidogenesis and it is mainly mediated by acute steroids-regulators. (StAR) [32, 33]. The physiological action of this protein depends on many internal factors such as PGF levels, COX-2 activity and exogenous factors by the overproduction of free radicals in a complex relationship still poorly understood [34–37]. StAR is also susceptible to a variety of pesticides as confirmed by Walsh et al. [2]. Steroidogenesis involves numerous steps in the synthesis of cholesterol to its transport within steroidogenic tissues and then its metabolism to steroids. Under normal physiological conditions, the collection of cholesterol by steroidogenic cells from free cholesterol circulating in the blood and high density / low density lipoprotein (HDL / LDL). In our previous studies, the administration of malathion at 200 mg / kg / day caused an imbalance in metabolic parameters marked by decreased plasma HDL cholesterol with elevated cholesterol, triglyceride and VLDL-CL levels [10]. Furthermore, malathion causes a decrease in the transport and use of cholesterol for steroidogenesis which explains this dyslipidemia. In addition, Barlow et al., [38] established that decreased cholesterol synthesis results in the downregulation of steroidogenesis. On the other hand, the subchronic LSEO co-administration considerably improves dyslipidemia induced by malathion in male mice. In addition, the rate of these parameters has been returned to near normal.

The decrease in mRNA expression of StAR, P450scc, 3ßHSD, and 17ß-HSD are previously shown to be responsible for cholesterol transport and testosterone synthesis in mice and rat testis [39]. In accordance with our results, Walsh et al. [2] demonstrated that dimethoate can inhibit steroidogenesis by blocking transcription of the StAR gene in MA-10 Leydig mouse tumor cells, thereby highlighting the sensitivity of StAR to environmental pollutants. In addition, Wang et al. [5] have demonstrated that the administration of cypermethrin during puberty has resulted in a disruption of testosterone synthesis by setting down the expression of the StAR gene. However, the overproduction of ROS observed on the malathion group causes mitochondrial membrane damage with a consequent imbalance in the level of StAR mRNA expression, inducing a reduction in cholesterol transport and testicular steroid formation in mice. In addition, the transport of cholesterol from the outer mitochondrial membrane to the inner membrane, the primary step of steroidogenesis, performed by the steroidogenic acute regulatory protein (StAR), is the mainly essential step in steroidogenesis [40].

As soon as it reaches the inner mitochondrial membrane, cholesterol and under the action of a key enzyme regulating steroidogenesis P450scc to produce pregnenolone [41] Our results showed that malathion induce a significant decrease of P450scc enzyme level. Fauser, showed the decrease in the level of P450scc can be attributed to a depletion of cholesterol availability that is due to a reduced level of FSH and LH [42]. In consequence, testosterone synthesis attenuation can be another results of decreased expression of P450scc enzyme. Recent studies in rats suggest that exposure to deltamethrin decreases steroidogenesis by directly inhibiting the expression of StAR and P450scc enzyme [43].

On the other hand, 3ß-HSD and 17ß-HSD, enzymes which can play a crucial role in testosterone biosynthesis. 3ß-HSD convert’s pregnenolone to progesterone, 17 hydroxypregnenolone to 17-hydroxyprogesterone (17-OHP), dehydroepiandrosterone (DHEA) to androstenedione, 17 ßHSD transformed androstenedione to testosterone in the smooth endoplasmic reticulum. A singnificant reduction of activity and expression these enzymes were also noted in malathion-treated mice compared to control group. In part, this reduction of 3ß-HSD and 17ß-HSD gene expression indicates a possible role of these genes to reduce testosterone level in male mice following malathion exposure, and in the other part, may be due also to the diminished expression of StAR. [41]. Ours results are consistent with and in agreement with other studies that have shown that exposure to malathion induces a disturbance of steroidogenesis and an imbalance of antioxidant enzymes [44].

We have been previously reported in our later studies that malathion induced oxidative stress in many organ systems such as liver [10], kidney [10], brain [45], and testis [3]. More importantly, we showed that LSEO significantly attenuated malathion-induced oxidative stress. Malathion-induced oxidative stress has been previously shown to be attenuated by many medicinal plant extracts such as *Olea europaea L.* However, we can suggest that our extract can attenuate the process of lipid peroxidation and/or antioxidant enzymes depletion implicated in the pathogenesis of malathion-induced reproductive damage.

On the other hand, using the DPPH radical-scavenging assay we showed that *L. stoechas* essential oils had an elevated scavenging capacity which may be correlated to the existence of phenolic compounds [46]. These letter, are the primal source of their antioxidant capacity, by scavenging free radicals as hydroxyl radical (OH^°^), which is the major cause of lipid peroxidation. However, its well known that sperm cells membranes are rich in polyunsaturated fatty acids and are very susceptible to free radical attack. Lipid peroxidation of sperm cell membranes is one type of cell damage induced by ROS, which causes an increase in membrane permeability, a disruption in respiratory chain and ATP production, as well as a decrease in phosphorylation of axonemal proteins. Malathion induced a decrease of the sperm quality might be due to the lessening of antioxidant enzyme activities and/or ROS accumulation. Free radical-induced sperm motility decrease was most probably due to a rapid loss of intracellular ATP, which altered axoneme arrangement and caused tail deformity [47].

Injury of cell DNA induced by ROS production led to the formation of some peroxidation products such as 8-oxo-7,8 dihydroxyguanosine, which cause disintegration and have a mutagenic consequence [48]. Higher DNA damage of sperm has been reported to reduce male fertility and antioxidants could be used to alleviate male infertility [49].

## Conclusions

We have evidently established that LSEO protect in opposition to malathion-induced steroidogenesis disruptions and oxidative stress in the reproductive function. This repro-protection offered by LSEO may be correlated in part to its antioxidant properties. This research supports the therapeutic potential of LSEO for prevention/treatment of reproductive-toxicity and should be further explored in clinical studies.

## Abbreviations

17 β HSD: 17 β-Hydroxysteroid dehydrogenase
3β HSD: 3 β-Hydroxysteroid dehydrogenase
CTR: Control group
FSH: Follicle-stimulating hormone
LH: Luteinizing hormone
LPO: Lipid peroxidation
LSEO: *Lavandula stoechas* Essential Oils
M: Malathion
MDA: Malonedialdehyde
OPs: Organophosphorus
OS: Oxidative stress
ROS: Reactive oxygenated species
StAR: Steroidogenic acute regulatory protein
TBARS: Thiobarbituric acid-reactive substances
WHO: World Health Organization
